# Supplementary material for: Can technology-based gait training result in relevant changes of ambulatory function in people with chronic, neurological diagnoses? A longitudinal, cohort study
Source: PLoS One. 2025 May 27;20(5):e0324062. doi: 10.1371/journal.pone.0324062 (PMC12111633; doi:10.1371/journal.pone.0324062)
Supplement: Attachment — Submitted filename: Response to Reviewers.pdf [file pone.0324062.s003.pdf]

Detailed point-by-point response to comments made by the editors and reviewers

We would like to thank the editorial board and the reviewer for their much-appreciated comments on our work “Can technology-based gait training result in relevant changes of ambulatory function in people with chronic, neurological diagnoses? A longitudinal, cohort study” submitted to PLOS ONE.

In the following letter, we directly respond to the comments and highlight where we adapted our revision to address these. To facilitate discussion of the concerns raised, we have decided to order the comments according to their point of reference in the paper, i.e., from the title to the end of the paper. We respond to each concern as outlined in the example below.

Topic

**R1**	Comments kindly provided by Reviewer 1
**E**	Comments kindly provided by the Editor
**Re-sponse**	Our response to the above comments
**Adap-tations**	List of adaptations we made in response to the above comments

Specific Comments

Methods

**R1**	27 participants completed three months while 20 completed six months of training. Why the unequal duration of trainings among your study participants. This may have a serious consequence on the other outcomes of the research.
**Re-sponse**	Thank you for this comment. We explain the reasons for the drop-outs in the limitations section starting on line 347. The linear mixed-effects model is accounting for the difference in duration of training to ensure that the results can be interpreted despite this.
**Adap-tations**	We added a statement about how the difference in duration of training has been accounted for in the statistical approach on line 355 of the revised manuscript with Track Change.

**R1**	VAMED Rehacenter Zurich Seefeld's former location<br>I am worried that this study was conducted in the premises of a company responsible for the manufacturing of medical equipment. I will need an explanation as to why conflict of interest is not a serious concern of the present study.
**Response**	You raise a valid concern. Yes, the former location of VAMED rehacenter Zurich Seefeld was in the premises of a medical device manufacturer (Hocoma AG). This has influenced which devices were placed in the rehabilitative center. These devices are very common in many rehabilitative centers for neurorehabilitation. Only devices that obtained certification as medical product and were available on the market were placed on the rehabilitative center. In addition, the center used equipment from other manufacturers as long as there was no similar equipment in the Hocoma portfolio.<br>For the study, the devices used during standard rehabilitation in the center were used. Hocoma AG was not involved in any way in the design, data collection, analysis of data storage of this study. The patient operations were managed by Rehaklinik Zihlschlacht AG, a subsidiary of VAMED Management und Service Schweiz AG, and have no legal connections to Hocoma AG.<br>The Rehacenter was a subtenant in the premises of Hocoma AG, which was regulated by a contract. No further connections between the Rehacenter and Hocoma AG existed at that time.
**Adaptations**	No adaptations to the manuscript were made.

**E**	Please ensure that your manuscript meets PLOS ONE's style requirements, including those for file naming.
**Response**	n.a.
**Adaptations**	The manuscript has been adapted to the style requirements.

**E**	Please note that PLOS ONE has specific guidelines on code sharing for submissions in which author-generated code underpins the findings in the manuscript. In these cases, we expect all author-generated code to be made available without restrictions upon publication of the work. Please review our guidelines at https://journals.plos.org/plosone/s/materials-and-software-sharing#loc-sharing-

	code and ensure that your code is shared in a way that follows best practice and facilitates reproducibility and reuse.
**Re-sponse**	We will share all author-generated code utilized for data analysis on Zenodo after acceptance of the manuscript.
**Adap-tations**	No adaptations to the manuscript were made.

**E**	Thank you for stating the following financial disclosure:<br>“This research did not receive funding from a funding agency. The cost for the training were partially covered by health insurances Sanitas and SWICA Health Insurance Ltd.”<br>Please state what role the funders took in the study. If the funders had no role, please state: "The funders had no role in study design, data collection and analysis, decision to publish, or preparation of the manuscript."<br>If this statement is not correct you must amend it as needed.<br>Please include this amended Role of Funder statement in your cover letter; we will change the online submission form on your behalf.
**Re-sponse**	We are happy to specify the financial disclosure statement:<br>“The funders consulted during the development of the study design to ensure that it met their funding principles. The final decision on the study design was made independently of the funders by the study team. The funders had no role in data collection and analysis, decision to publish, or preparation of the manuscript.”
**Adap-tations**	The amended Role of Funder has been included in the cover letter

**E**	We note that you have indicated that there are restrictions to data sharing for this study. PLOS only allows data to be available upon request if there are legal or ethical restrictions on sharing data publicly. For more information on unacceptable data access restrictions, please see http://journals.plos.org/plosone/s/data-availability#loc-unacceptable-data-access-restrictions.<br>Before we proceed with your manuscript, please address the following prompts:<br>a) If there are ethical or legal restrictions on sharing a de-identified data set, please explain them in detail (e.g., data contain potentially identifying or sensitive patient information, data are owned by a third-party organization, etc.) and who has imposed them (e.g., a Research Ethics Committee or Institutional Review Board, etc.). Please also provide contact information for a data access

	committee, ethics committee, or other institutional body to which data requests may be sent.<br>b) If there are no restrictions, please upload the minimal anonymized data set necessary to replicate your study findings to a stable, public repository and provide us with the relevant URLs, DOIs, or accession numbers. For a list of recommended repositories, please see https://journals.plos.org/plosone/s/recommended-repositories. You also have the option of uploading the data as Supporting Information files, but we would recommend depositing data directly to a data repository if possible.<br>We will update your Data Availability statement on your behalf to reflect the information you provide.
**Response**	We will make our de-identified data available via a data repository (Zenodo) after acceptance of the manuscript.
**Adaptations**	No adaptations to the manuscript were made.

E	When completing the data availability statement of the submission form, you indicated that you will make your data available on acceptance. We strongly recommend all authors decide on a data sharing plan before acceptance, as the process can be lengthy and hold up publication timelines. Please note that, though access restrictions are acceptable now, your entire data will need to be made freely accessible if your manuscript is accepted for publication. This policy applies to all data except where public deposition would breach compliance with the protocol approved by your research ethics board. If you are unable to adhere to our open data policy, please kindly revise your statement to explain your reasoning and we will seek the editor's input on an exemption. Please be assured that, once you have provided your new statement, the assessment of your exemption will not hold up the peer review process.
**Response**	Data has been prepared for publication. We will make our data available via a data repository (Zenodo) after acceptance of the manuscript.
**Adaptations**	No adaptations to the manuscript were made.

E	Please include your full ethics statement in the ‘Methods’ section of your manuscript file. In your statement, please include the full name of the IRB or ethics committee who approved or waived your study, as well as whether or not you obtained informed written or verbal

	consent. If consent was waived for your study, please include this information in your statement as well.
**Re-sponse**	We added the study ID provided by the responsible ethics committee in the “Methods” section of the manuscript
**Adap-tations**	Study ID added on line 107 of the revised manuscript with Track Change.

E	Please include captions for your Supporting Information files at the end of your manuscript, and update any in-text citations to match accordingly. Please see our Supporting Information guidelines for more information: http://journals.plos.org/plosone/s/supporting-information.
**Re-sponse**	n.a.
**Adap-tations**	The captions for the supporting files were added at the end of the manuscript and the files were re-uploaded with the respective file name. The documents were also cited within the text on line 107/108 of the revised manuscript with Track Change.

E	Please review your reference list to ensure that it is complete and correct. If you have cited papers that have been retracted, please include the rationale for doing so in the manuscript text, or remove these references and replace them with relevant current references. Any changes to the reference list should be mentioned in the rebuttal letter that accompanies your revised manuscript. If you need to cite a retracted article, indicate the article’s retracted status in the References list and also include a citation and full reference for the retraction notice.
**Re-sponse**	The reference list has been verified.
**Adap-tations**	The language of the reference list has been set to English.
