# Supplementary material for: Can technology-based gait training result in relevant changes of ambulatory function in people with chronic, neurological diagnoses? A longitudinal, cohort study
Source: PLoS One. 2025 May 27;20(5):e0324062. doi: 10.1371/journal.pone.0324062 (PMC12111633; doi:10.1371/journal.pone.0324062)
Supplement: Attachment — Submitted filename: Reviewer One.doc [file pone.0324062.s001.doc]

**Reviewer One**

Response from authors is required: 27 participants completed three months while 20 completed six months of training. Why the unequal duration of trainings among your study participants. This may have a serious consequence on the other outcomes of the research.

VAMED Rehacenter Zurich Seefeld’s former location

I am worried that this study was conducted in the premises of a company responsible for the manufacturing of medical equipment. I will need an explanation as to why conflict of interest is not a serious concern of the present study.
